# The Living Transcendental — An Integrationist View of Naturalized Phenomenology

**DOI:** 10.3389/fpsyg.2020.01548

**Published:** 2020-07-14

**Authors:** Thomas Netland

**Affiliations:** Department of Philosophy and Religious Studies, Norwegian University of Science and Technology, Trondheim, Norway

**Keywords:** naturalized phenomenology, Merleau-Ponty, transcendental philosophy, Schneider, enactivism

## Abstract

In this article I take on the “Transcendentalist Challenge” to naturalized phenomenology, highlighting how the ontological and methodological commitments of Merleau-Ponty’s philosophy point in the direction of an integration of the transcendental and the scientific, thus making room for a productive exchange between philosophy and psychological science when it comes to understanding consciousness and its place in nature. Discussing various conceptions of naturalized phenomenology, I argue that what I call an “Integrationist View” is required if we are to make sense of the possibility of productive exchange between phenomenology and the sciences. My main argument is that if we conceive of consciousness as a structure of behavior ontologically prior to the distinctions between objectivity and subjectivity and third- and first-person perspectives, we arrive at a view of the transcendental as not essentially separate from the domain of science, but rather as contingent organizational norms of empirical nature that are best illuminated through a dialectical exchange between phenomenological and scientific approaches. I end by showing how Merleau-Ponty’s engagement with the “Schneider case” in an example of such an integration.

## Introduction

During the last decades, phenomenology has become increasingly influential as a resource for developments in the mind sciences. This is especially so in the research program known as “the enactive approach,” one of the central tenets of which is, in the words of its cofounder Evan Thompson, that “[i]t is not only possible, but also necessary, to pursue phenomenology and experimental science as mutually constraining and enlightening projects” (2007, p. 273). The prospects of such a relationship is, however, not without difficulties, but has been challenged both by people skeptical of phenomenology’s credentials altogether and by phenomenologists who reject the idea of “naturalizing” a philosophy that, in their view, is concerned with the conditions that *enable* scientific thinking in the first place and as such cannot be informed by its results. This latter, “Transcendentalist Challenge” to naturalized phenomenology, is the motivating force for this paper.

My overarching aim in what follows is to propose the position I call the “Integrationist View” (IV), which consists in a reconceptualization of the notions of the “transcendental” and “nature” in a way that allows for a methodological and ontological integration of scientific and phenomenological perspectives. In outlining this view, I draw on the early works of Maurice Merleau-Ponty, the classical phenomenologist known for his extensive engagement with scientific literature. I am far from the first to argue that Merleau-Ponty’s philosophy is a promising starting point for making sense of the project of naturalizing phenomenology. At the same time, as Jack Reynolds has recently observed, “exactly how to understand the Gordian knot concerning Merleau-Ponty’s implicit and explicit commitments regarding transcendental reasoning, phenomenology and empirical science, remains contested, more than 50 years after his death” (2017, p. 85). Thus, although I do not presume to completely resolve this knot here, this paper is also a contribution to discussions in Merleau-Ponty scholarship. The reading I propose emphasizes the significance of his first book, *The Structure of Behavior* (1942/1963; henceforth *Structure*), as a background for making sense of the further development of his thought. As such, my reading is at least partly aligned with and indebted to Toadvine (2009) and Morris (2018), both of which, notwithstanding some interpretative differences, see Merleau-Ponty primarily as a philosopher of nature, one of the key concerns of which was to establish the idea of an immanent, expressive *sense* of nature in the form of the embodied and active structure of living organisms’ existence. In this way, I see this paper as a contribution to the project of construing enactivism as a philosophy of nature (e.g., Gallagher, 2017, pp. 21–24).

The crux of my argument is that if we conceive of consciousness as a structure of behavior ontologically prior to the distinctions between objectivity and subjectivity and third- and first-person perspectives, we arrive at a view of the transcendental as *not* essentially separate from the scientific, but rather as contingent organizational norms of nature that are best illuminated through a dialectical exchange between phenomenological and scientific approaches. I start by sketching the general contours of transcendental philosophy and the “Transcendentalist Challenge” to naturalized phenomenology, taking Gardner’s (2015) transcendentalist reading of Merleau-Ponty as the point of departure (1). I then turn to Zahavi’s (2017) suggestion of two alternative ways to understand what a “naturalized phenomenology” amounts to, arguing that the position I label “Modest Transcendentalism” lacks the resources for making adequate sense of the possibility of a productive exchange between phenomenology and science, and propose that this task rather requires the “Integrationist View” (2). Thereafter, I show how the notion of structures of behavior is apt to yield an integrationist ontology (3) before I return to criticize Gardner’s transcendentalist reading of Merleau-Ponty in the context of the phenomenological method (4). Lastly, I propose a way to read Merleau-Ponty’s engagement with the “Schneider case” in *Phenomenology of Perception* (1945/2012; henceforth *Phenomenology*) as an instance of the IV in action (5).

## The Transcendentalist Challenge and Varieties of Transcendentalism

In “Merleau-Ponty’s Transcendental Theory of Perception,” (2015) Gardner gives expression to one of the main theoretical challenges to the idea of a naturalized phenomenology, namely, the argument that phenomenology is essentially a form of *transcendental* philosophy and, as such, operates in a domain strictly independent from the scientific.^1^ Indeed, the main target of Gardner’s paper is what he calls the “Psychological Interpretation,” which reads Merleau-Ponty’s *Phenomenology* as offering insights about perception that can both be put to use by and find support in empirical cognitive science.^2^ On Gardner’s reading, on the contrary, Merleau-Ponty’s arguments have the same “form and idealistic trajectory” as Immanuel Kant’s transcendental philosophy (2015, p. 313) and “involves no positive estimate of psychological science as an independent source of knowledge that philosophy ought to accommodate” (2015, p. 319), leading him to conclude that the naturalistic philosophy of psychology that some find in the *Phenomenology* “has only an oblique relation to the position Merleau-Ponty is actually arguing for” (2015, p. 321). Before looking closer at Merleau-Ponty’s position, let us have a look at transcendental philosophy more generally.

What is transcendental philosophy? The history of this notion and the discussions surrounding it shows that it is difficult, if not impossible, to give one precise characterization that covers all its appearances.^3^ I will try to give a sense of this fluidity of the notion in what follows, but take this as a first, provisional definition: Transcendental philosophy aims to uncover the ground for objective knowledge, where “ground” is understood not as a Cartesian foundational proposition that secures the possibility of knowledge, but rather as the structures of consciousness constitutive of our knowing. The prime example here is Kant. In *Critique of Pure Reason* (2007), he asked how it is possible for experience to be a source for knowledge and answered that the necessary conditions for this is that experience be oriented in space and time (the forms of intuition), structured in conformity with the categories of the understanding (e.g., causality and substantiality), and unified in relation to the unity of the transcendental subject (the “I think”).^4^ Notice how this project is fundamentally different from what we find in the sciences. After all, science takes the possibility and validity of experience, objectivity, and knowledge for granted, depending on these in its project of gathering facts and constructing theories about the world. Transcendental philosophy, on the other hand, does not seek fact or theory in the same sense but rather the conditions that make them possible. We can thus see how the idea of a productive exchange between these two domains is problematic: science does not seem to require an understanding of its transcendental conditions of possibility in order to succeed, and transcendental philosophy cannot rely on scientific findings without presupposing what it seeks to understand.^5^

This distinction is underlined by the fact that transcendental philosophy is a non-empirical, *a priori* endeavor. Consider how Kant deals with the concept of causality: On his view, we do not acquire this concept through experience; rather, it belongs to the subject as one of its necessary *conditions* for experience to be possible in the first place. Methodologically, this means that the transcendental here is identifiable by purely *a priori* means. Exactly how to understand the nature of Kant’s transcendental arguments is a discussion in its own right and not something I will dig into here.^6^ For our purposes, it suffices to draw attention to one way we can understand the contrast and continuity between Kant and the phenomenological tradition when it comes to the notion of the transcendental. In this context, we can distinguish between two forms of transcendental argument found in Kant—one that is dismissed by the phenomenologists and one that they to some degree take up and refine. In the former, we find progressive arguments aimed at establishing the necessary objective validity of certain concepts (e.g., causality). These lead Kant to construe the transcendental as structures belonging to subjectivity (more precisely to the understanding) independently of any particular experience, which determines in advance the possible form of all future experience. The latter form of argument is regressive, beginning from given facts or experiences and proceeding to reconstruct the conditions for the possibility of their givenness as such.^7^ This means that one here is paying more attention to concrete matters and how these are experienced and apprehended compared to in the former case, where the aim rather is to establish the necessary forms all such matters must conform to.

By rejecting the first kind of argument and modifying the second, Edmund Husserl’s transcendental phenomenology represents a shift in the notion of the transcendental. Now, the transcendental is conceived not as belonging solely to the subject but to the subject-world *correlation*, not as principles abstractly outlining the form of all possible experience but as structures constitutive of and originating within actual experience.^8^ This makes the necessity and a prioricity of the transcendental in Husserl’s philosophy quite different from that found in Kant. As Julia Jansen observes (presumably thinking primarily of Kant’s progressive arguments),

Kant thinks of a necessary unity as a unity that receives its necessity “top–down” from the “highest point” of reason […]. Husserl, on the contrary, thinks of unity “laterally,” as a unity of “coincidence (*Deckung*),” which enables *a priori* insight not only into necessities that “reason itself produces according to its own plan” (B xiii), as Kant famously claimed, but also into necessities reason genuinely *discovers* (Jansen, 2015, pp. 48–49, emphases in original).

In other words, the transcendental is now understood as in a certain sense experientially discoverable, drawing it closer to the empirical domain. This is evident in Husserl’s claim that the proper method of transcendental philosophy should be *description* rather than deduction. On his view, this shift represents a necessary correction of Kant’s project, which from the phenomenological perspective takes the form of problematic metaphysics, resting for instance on a misguided separation between sensibility and the understanding (ibid., p. 59). Rather than assuming such a separation and then attempting to identify the contribution of each faculty through a rational construction, phenomenology takes the actuality of perception as its point of departure and seeks to describe, clarify, and analyze the emergence of meaning and objectivity as evident *therein*.

While this surely moves the transcendental domain closer to the empirical relative to what we find in Kant, it does not entail that the distinction between the transcendental and scientific domains collapses. In Husserl’s phenomenology, the key methodological tools for arriving at the domain proper to transcendental phenomenology are the *epoché* and the *reduction*. The *epoché* amounts to a shift from the “natural” to the “phenomenological” attitude through *bracketing* or *suspending* our normal interest in and presuppositions regarding the external world as such, so as to focus on the subject-world correlation—i.e., on the *how* of experience rather than the *what* of the experience*d*. The reduction is then the next step, consisting in the systematic examination of this correlational structure in light of its transcendental function.^9^ This now marks the difference between phenomenology (*qua* transcendental) and science. While they both might take their data from experience, their attitudes are fundamentally distinct—the latter seeks to know the *objects* of experience and takes their existence as such for granted, whereas the former aims to clarify the constitutive structures of the givenness of the world thanks to which it appears as objective, meaningful, etc. In *Rethinking Transcendentalism: the Limits of Transcendental Reflection*, we will look closer at Merleau-Ponty’s verdict of this method, which famously is that “the most important lesson of the reduction is the impossibility of a complete reduction” (2012, p. lxxvii).

The transcendental conditions identified through the phenomenological method are of a quite different sort from those deduced by Kant. Here, we move from the form-imposing role of the categories of the understanding, to constitutive conditions “visible” within experience, such as consciousness’ horizonal structures (its co-intention of “absent” and indeterminate features such as past and future, the hidden profiles of visual objects, etc.). Again, this means that we are operating with a quite different notion of “transcendental” here than what we started out with. As Jansen suggests, Kant would probably have dismissed the transcendental structures identified by Husserl as “crude empirical generalities” (2015, p. 78) that fails to meet his strict criteria for *a priori* necessity. As we will see in the next section, some of these “generalities” identified by phenomenology—more precisely, the invariances of experience disclosed by eidetic analyses^10^ —seems to mark a point of contact between phenomenology and psychological science on Husserl’s view. A discussion of what this means for the prospects of a naturalized phenomenology will have to wait until then.

For now, these are the key takeaways. Despite his departure from Kant’s method and metaphysics, Husserl maintains the distinction between transcendental and scientific enterprises. However, the transcendental is now understood as “closer” to the empirical, giving a new sense to its necessary and *a priori* status (invariant/essential constitutive structures *of* experience rather than forms logically imposed upon it). The distinction between science and transcendental philosophy is maintained but now understood as one of *attitudes*. The prospects for a mutually informative relation between them still looks dim—after all, one presupposes the attitude which the other suspends and analyses, and more generally, they are simply preoccupied with different kinds of questions.

Let us now return to Gardner’s transcendentalist interpretation of Merleau-Ponty’s *Phenomenology*. On his view, transcendental philosophy here undergoes yet a transformation. That is, he sees Merleau-Ponty as establishing *preobjective* perception as “a ground-level transcendental condition” (2015, p. 307). As such, Merleau-Ponty’s philosophy entails a critique of both Kant and Husserl’s transcendental projects to the extent that they are characterized by “objective thought.” “Objective thought” here means a certain dogmatic way of accounting for experience’s “articulation into objects and its character […] as involving a relation of subject to object” (ibid, p. 301). The intellectualist tendencies of Merleau-Ponty’s transcendentalist predecessors fall into this category due to their taking thoughts about objects as the ultimate *explanans* (i.e., objective thought is responsible for the objectual character of experience). A different form of the same dogma is shared by the view we can call scientific realism or naturalism (“empiricism” in Merleau-Ponty’s terminology), which takes the objectual character of experience to be caused by a subject-independent world already articulated into objects. In short, both intellectualism and empiricism take objectivity as a *given*, and Merleau-Ponty’s transcendental project consists in disclosing the *origin* of objective thought as such from the preobjective and ambiguous perceptual field. It is on this basis that Gardner dismisses interpretations of Merleau-Ponty that see him as providing, among other things, “arguments for the dependence […] of consciousness on embodiment [and] a convincing critique of the representationalism which holds sway in cognitive science” (2015, p. 297). Such psychological readings take consciousness and perception as *objects* to be described and explained, rather than as the field where objectivity emerges in the first place, and thus leaps over the issue that actually drives the *Phenomenology*.

Although I disagree with the strict separation Gardner sets up between transcendentalism and science, I think his claim that Merleau-Ponty’s transcendental philosophy is *ontological* in form is basically correct.^11^ As he says, “talk of pre-objective being is not just talk of *experience* prior to the involvement of objectivity concepts in experience: it is talk of experienced *being* which is pre-objective” (2015, p. 298, emphases in original). The *Phenomenology*’s critique of “objective thought,” then, is not merely a critique of certain conceptions of *experience*; rather, it is “a critique of the *metaphysical* claim that objective representation is adequate to the representation of reality or, put the other way around, that reality is as objectivity concepts represent it as being” (ibid.). As we will see later, this ontological dimension of Merleau-Ponty’s project is a key element in the Integrationist View I am suggesting. In other words, my point of divergence from Gardner’s interpretation concerns what the metaphysics of preobjectivity entails for the prospects of a mutually enlightening relation between phenomenology and science. On my view, which conceives it as a continuation of the project *Structure* sets in motion, Merleau-Ponty’s ontology represents a promising step toward a “phenomenologizing” of nature where the border between the transcendental and the scientific becomes diffused. For Gardner, on the contrary, the transcendental nature of Merleau-Ponty’s project means that its extensive engagement with scientific literature must be understood merely as, in Reynolds’ apt words, a sort of “Wittgenstein’s ladder,” which should be kicked away once the transcendental is reached (2017, p. 98). “Engagement with scientific psychology,” Gardner claims,

sharpens and refines our appreciation of psychological considerations, which in turn helps us to reach a position from which phenomenological truth can be grasped on the basis of an apodictic relation to the pre-objective, rendering transcendental reflection strictly independent of any application of the scientific method (Gardner, 2015, p. 319).

The idea here is that considerations of psychological science might serve the instrumental role of ridding transcendental philosophy of its intellectualist pretensions, but that transcendental reflection proper gets underway only after this labor is done with and then within an autonomous domain indifferent to the scientific. On this point, then, Merleau-Ponty appears to be fully in line with his transcendentalist predecessors. “Merleau-Ponty,” Gardner says, “provides […] many statements of how the conditions that his phenomenology uncovers are intended to be in the true and genuine sense transcendental, i.e., *a priori* and necessary, and non-identical with empirical, contingent, or mundane states of affairs” (2015, p. 300). A legitimate question here, however, is how it is possible for transcendental philosophy to be both *reformed* by (ibid, p. 319) *and* “strictly independent of” considerations from scientific psychology. Can one really have both?

Gardner’s insistence on a strict independence of the transcendental from the scientific seems to stem from a specific conception of what a mutually enlightening relation between them would have to amount to. According to him, naturalized interpretations of Merleau-Ponty’s project will not only have the consequence that his philosophy can “become subject […] to empirical correction” but also that “the task of explanation […] tends inevitably to pass out of the hands of phenomenology into neurophysiology and other more empirically tough-minded quarters” (2015, p. 297). In other words, it would ultimately amount to an unequivocal abandonment of transcendental philosophy in favor of an all-encompassing scientific naturalism, where all legitimate questions are seen as answerable by the methods of natural science. There surely are those who advocate this form of “naturalized phenomenology.”^12^ We find a prime example in the introduction to the anthology *Naturalized Phenomenology*, where the editors explicitly state that “naturalized” here means “integrated into an explanatory framework where every acceptable property is made continuous with the properties admitted by the natural sciences” (Roy et al., 1999, pp. 1–2). Now, if this is what one means by naturalization, and one by “phenomenology” refers to the philosophical tradition of Husserl and Merleau-Ponty, then the notion of a naturalized phenomenology is an impossibility—a category mistake (Zahavi, 2013, p. 30). After all, phenomenology thus conceived would have to partake without question in the “natural attitude” or “objective thought,” giving up on its defining task of clarifying the constitution and/or origin of objectivity and thus ceasing to be phenomenology altogether.

That being said, the choice Gardner seems to presuppose between, on the one hand, a transcendental philosophy indifferent to the results of science and, on the other, a naturalistic philosophy that yields all authority to such findings is a false dichotomy. As Zahavi (2013, 2017) suggests, there seems to be at least two alternative conceptions of what a naturalized phenomenology can amount to available. Below I present these, arguing that we should favor the alternative I label the Integrationist View over the more conservative Modest Transcendentalism before I, in the remainder of this text, propose a reading of Merleau-Ponty’s philosophy as an instance of the former.

## From Modest Transcendentalism to the Integrationist View

While Zahavi on multiple occasions has raised concerns over “naturalizing” approaches to phenomenology from a transcendentalist perspective (2004; 2010; 2011; 2013; and 2017), he is simultaneously one of our contemporary phenomenological philosophers that has done most to facilitate and engage in fruitful dialogs with psychological science. It is thus not surprising that we in his writings find suggestions for conceptions of naturalized phenomenology where the philosophical or transcendental core of phenomenology is maintained. Zahavi tends to point to two such alternatives. The first keeps the idea of phenomenology as transcendental philosophy where “transcendental” entails belonging to a domain strictly separate from the natural and scientific but is more liberal than Gardner’s view in that it, nonetheless, allows for some form of mutual enlightenment between the two domains (2017, pp. 162–163). The second alternative is based on rethinking the very notions of the “transcendental” and the “natural” as traditionally conceived, pushing for a tighter integration of phenomenology and science within the framework of a “phenomenologized nature” (2017, p. 167). I call these Modest Transcendentalism (MT) and the Integrationist View (IV), respectively.

Zahavi makes it clear that he is sympathetic to both alternatives and emphasizes that they “should not be seen as incompatible alternatives between which we have to choose” but that “they might be pursued simultaneously” (2017, p. 169). He argues convincingly that Husserl, despite his antinaturalist reputation, subscribed to MT and suggests that he might even have accepted the more radical IV (2017, p. 168). Thus, although I, in the following, use Zahavi’s reading of Husserl as representative of MT, I do not assume either of them to be unequivocally committed to this view. I will, however, dispute Zahavi at one account: his compatibility claim quoted above, which he makes without elaboration. How can the two alternatives be compatible? After all, IV aims to unsettle a core pillar of MT’s framework, namely, the separation of the transcendental from the natural. As long as this is what defines the difference between the two alternatives, it seems that we *do* have to choose between them. If that is right, I believe that IV has the stronger case. The reason for this is that, when pressed to make adequate sense of the relationship it sets up between phenomenology and science, MT seems to have difficulties preserving the traditional transcendental–natural distinction it presumably subscribes to and to inadvertently and implicitly collapse into a view more like IV. In other words, my argument in what follows is that IV is best suited to give weight to and make coherent the productive exchange between philosophical and scientific perspectives envisioned by MT.

What does the exchange between phenomenology and science consist in for MT? Zahavi suggests the following:

Phenomenology can question and elucidate basic theoretical assumptions made by empirical science, just as it might aid in the development of new experimental paradigms. Empirical science can present phenomenology with concrete findings that it cannot simply ignore, but must be able to accommodate (2017, p. 162).

Through its eidetic analyses of consciousness, phenomenology yields descriptions and theories of phenomena such as perception, imagination, embodiment, etc., which can serve as basis for engaging critically with scientists’ assumptions regarding the same phenomena.^13^ Notice that we, in the quote’s second sentence, find a clear contrast to the view offered by Gardner’s Transcendental Interpretation of Merleau-Ponty, which, remember, rejects the idea of science as “an independent source of knowledge that philosophy ought to accommodate” (2015, p. 319). The question now is, how does this relationship suggested by MT square with the idea that phenomenology and science belong to two essentially separate domains? That is, *in virtue of what* is phenomenology justified as having a say concerning scientific theory, and empirical findings an impact on phenomenology?

MT’s first response is that we here are not yet talking about *transcendental* phenomenology. Zahavi reminds us of Husserl’s view that “to engage in an eidetic and *a priori* analysis of experiential consciousness is to do psychology—and not yet phenomenology proper” (2017, p. 157). MT’s commitment to a separation of the transcendental and the scientific together with its opening for a relationship of mutual enlightenment between phenomenology and science thus seems to rest upon a distinction between two forms of phenomenology—transcendental and psychological. Here, the latter is understood as remaining within the natural attitude, studying consciousness for its own sake in a non-reductive way, whereas the former is interested in consciousness “insofar as [it] is taken to be a condition of possibility for meaning, truth, validity, and manifestation” (ibid.). At this point, it can seem as if MT’s solution to how phenomenology and science can cooperate is simply to define phenomenology in this context as a non-transcendental enterprise. If that were the case, it would arguably not be a solution as much as a case of moving the goalposts. What we are after is, after all, a way to naturalize phenomenology that does not simply neglect or erase its philosophical credentials.

MT avoids this objection by pointing to the intimate connection between transcendental and psychological phenomenology, making the latter more of a mediator than a substitute for the former in the envisioned phenomenology-science exchange. Although different from transcendental phenomenology in that it remains within the natural attitude, investigating consciousness as a region of the objective world rather than as a condition of possibility for that world, phenomenological psychology has the potential to *lead to* transcendental phenomenology if pursued in a radical and precise enough manner (ibid., p. 157). In approaching a comprehensive understanding of consciousness as non-reduced phenomenon, phenomenological psychology will eventually be prompted to acknowledge consciousness’ transcendental significance. In other words, it seems that the line between psychological and transcendental approaches to consciousness is not so easy to draw after all. On the contrary, on this view, “psychology qua the study of consciousness *contains a transcendental dimension* and is ultimately *part of* transcendental philosophy” (Zahavi, 2017, p. 159; my emphases). This connection between the transcendental and the psychological is also acknowledged by Merleau-Ponty, who states that “the transcendental attitude is already implied in the psychologist’s descriptions” (2012, p. 60), even going so far as to label the relationship one of “interpenetration” and “mutual envelopment” (1964, p. 73). The question for MT, however, is how it can subscribe to this way of understanding the relationship between phenomenology and science without sacrificing any of its other commitments. That is, while the transcendental–psychological connection sketched here surely makes more sense of the possibility of mutual enlightenment between transcendental phenomenology and science, it simultaneously hints at a diffusion of the border between the two—a border MT is supposed to leave unquestioned. What does it mean to let “the very conceptions of naturalism and transcendental analysis remain unaffected” (Zahavi, 2017, p. 163) in light of these considerations?

This tension seems to only become more pressing upon further interrogation of this view. Let me draw attention to three points that illustrate this, and which I believe pulls MT closer to IV. First, a possible objection to the view that a productive exchange between phenomenology and science is possible is that there is a mismatch between the *a priori* status of transcendental reflection and the *a posteriori* nature of empirical findings. How can *a priori* insights inform *a posteriori* sciences, or vice versa? Zahavi responds to this by drawing attention to Husserl’s view that *a priori* phenomenological insights are not immune to corrections in light of new evidence, but rather “always possess a certain provisionality, a certain presumptiveness” (2017, p. 155). “Our *a priori* knowledge,” he elaborates, “is, in short, fallible; if we come across putative empirical counterexamples to our alleged eidetic insights, they need to be taken seriously and cannot simply be dismissed as irrelevant” (ibid.). What we have here, then, is a view of the phenomenological *a priori* not only as *fallible* but as potentially challengeable by *empirical* findings. Now, this idea of revisable transcendental insights is prepared already by what we saw in the previous section regarding the regressive nature of phenomenological transcendentalism. After all, if the task is to start from actual experiences and clarify their constitutive structures, then discoveries that prompt revisions of one’s earlier articulations are always a possibility. Still, it is not clear how scientific findings can work as counterexamples to eidetic insights on MT’s model.

Second, it is important to note that while the prime example of science in MT’s model of phenomenology–science cooperation is phenomenological psychology, which is concerned with a non-reductive understanding of consciousness and takes first-person experience as its point of departure (Zahavi, 2017, pp. 157, 159), this does not mean that “empirical findings” in this context is limited to descriptions of first-personal consciousness as such. Among the empirical sciences that Zahavi mentions as most promising for engaging in productive exchange with phenomenology, we find disciplines such as anthropology, psychopathology, and developmental psychology (2017, p. 152)—all of which, notwithstanding their non-reductive, person-directed nature, at least in part rely on third-personal observations of bodily behavior and its worldly (material and cultural) conditions. Hence, if it is right that findings in these domains “might be taken up by, and consequently influence or constrain, an analysis of transcendental subjectivity” (ibid, pp. 159–160), we need a way to make sense of how third-person perspectives can play this role.

Third, for this transcendental–psychological exchange to work, one must at least admit that the two domains are dealing with subject matters that are closely enough related for them to be relevant to each other. This is staunchly rejected by Gardner, who insists on an “absolute, non-epistemological distinction” between the phenomenal and the objective body (2015, p. 298). This distinction, however, seems to be put into question by Zahavi’s Husserl-inspired MT:

the relation between the transcendental subject and the empirical subject is for Husserl not a relation between two different subjects, but between two different self-apprehensions. The transcendental subject and the empirical subject is but one subject, though viewed from different perspectives. The transcendental subject is the subject in its primary constitutive function. The empirical subject is the same subject, but now apprehended and interpreted as an object in the world (2017, p. 158).

According to MT, then, the separate domains of phenomenology and psychological science are just two different ways of approaching the same subject. While this undoubtedly helps make sense of the relation between the transcendental and the psychological sketched above, it also seems call for a philosophical framework beyond what MT offers. That is, how can a proposal in which “nothing […] entails or necessitates the need for a more fundamental rethinking of the relation between the constituting and the constituted” (ibid, p. 163) make room for the idea that the (constituted) empirical subject is the *same* as the (constituting) transcendental subject?^14^ One would think that, without a fundamental rethinking of this relation, the two subjects could *not* be the same, since they would always find themselves at opposite poles of the constitutive correlation. At the very least, the idea of identity between the two subjects would not represent the solution to a problem as much as a problem in itself, as Husserl acknowledged when he in *The Crisis of the European Sciences and Transcendental Phenomenology* described what has come to be known as “the paradox of subjectivity”^15^ : “The difference between empirical and transcendental subjectivity remained unavoidable; yet just as unavoidable, but also incomprehensible, was their identity. I myself, as transcendental ego, ‘constitute’ the world, and at the same time, as soul, I am a human ego in the world” (1970, p. 202). In that work, Husserl’s way out of the paradox seems to have been, in Anthony Fernandez’ words, “a complete dehumanizing and decontextualizing of the transcendental ego” (2015, p. 294), thus ultimately denying the identity between the empirical and transcendental subject after all. “In the epoché and in the pure focus upon the functioning ego-pole […],” Husserl says, “*nothing human is to be found*, neither soul nor psychic life nor real psychophysical human beings; all this belongs to the ‘phenomenon,’ to the world as constituted pole” (1970, p. 183; my emphasis). I am not saying that MT necessarily is committed to accept this consequence, but it surely highlights the difficulty of stating the identity of the transcendental and empirical subject within this more conservative transcendentalist position.

Putting together the above considerations, we get a view that says that scientific approaches to consciousness have a transcendental dimension, that *a priori* transcendental analyses are vulnerable to change in light of third-personal empirical evidence, and that the transcendental and empirical subject ultimately is the *same* subject. As we have seen, these features of MT seem to put pressure on its commitment to preserve the classical notions of “transcendental” and “natural.” What is missing here seems to be precisely what is offered by our second alternative, the Integrationist View: a model of how the transcendental and empirical aspects of consciousness are *integrated*, so as to make adequate sense of a mutually enlightening relationship between phenomenology proper and science.

As mentioned, one central feature of this view is the aim to rethink the concept of nature in a “phenomenologized” fashion. Of course, “nature” never had a clear and uncontroversial meaning in the first place, so what concept is it more specifically that we are asked to rethink here? Briefly put,^16^ it is the *objectivist* concept, which neglects that objects are always accessed by a subject and moreover eliminates anything that is assumed to be mere “products” of human subjectivity (meaning, quality, normativity, etc.) from its picture of the real.^17^ While classical transcendentalism tends to be critical of “expansionist” forms of objectivism that purports to shape all forms of thought in its own image, it has generally left objectivist naturalism untouched insofar as it is understood to be a necessary presupposition for the sciences. The assumption that all of natural science is committed to such a position is at least part of the reason for transcendental phenomenologists’ long-standing insistence on operating in an autonomous intellectual domain. IV, in contrast, calls for an uprooting of this view of nature altogether, toward one able to incorporate consciousness’ transcendental status and the reality of phenomena such as subjectivity, meaning, and normativity. Zahavi points to Thompson’s *Mind in Life* as the “currently most comprehensive attempt” at developing such a view (2017, p. 164). In his own words, Thompson’s project starts from “a recognition of the transcendental and hence ineliminable status of experience” and aims toward “a different kind of approach to matter, life, and mind from objectivism and reductionism” (2007, p. 87). Central to this approach is the thesis that there is a *deep continuity* pertaining to the organizational structures of mind and life (2007, pp. 128–129).^18^

This leads directly to IV’s second defining trait—the transformation of transcendental philosophy from an isolated to a more pluralistic and cooperative enterprise. Where IV’s rethinking of nature consists in making room for the constitutive organization of structures of meaning and subjectivity as natural phenomena, its rethinking of the transcendental consists in understanding transcendental reflection as *part of* and directed at nature thus conceived. To make this more concrete, consider how *embodiment* is both a transcendental condition for our openness to the world *and* entails biological existence. (As such, it is a crystallization of the paradox of subjectivity). From a more traditional perspective, the transcendental and the biological would seem to be completely unrelated. IV’s conceptual transformations, however, holds the promise of a comprehensive ontology upon which these might be seen as mutually enlightening and constraining perspectives. Zahavi’s presentation of this trait of IV seems to point in the same direction. For instance, he cites the suggestion of Roy et al. (1999, p. 61) that “Husserl’s and Merleau-Ponty’s investigations of the lived body focus on a locus where ‘a *transcendental analysis and a natural account are intrinsically joined*”’ (2017, p. 164). Furthermore, he draws attention to Merleau-Ponty’s call for us to “search for a dimension that is beyond both objectivism and subjectivism” where we would not have to “choose between an external scientific explanation or an internal phenomenological reflection” (ibid, p. 165). This dimension, I will try to show below, is in Merleau-Ponty’s first works illuminated through the notion of structures of behavior and—pace Gardner—the preobjective perceptual field. As we will see, this rethinking of transcendental philosophy comes with two significant adjustments relative to its previous form: a step away from first-personal phenomenology, in the sense that the constitution of givenness is no longer an act that is manifest only to the subject of the given, and a recognition of the significance of contingency for its project.

In the remainder of this paper, I will try to show how Merleau-Ponty, despite Gardner’s claims to the contrary, offers a promising starting point for developing the IV.^19^ An important reason for why this is not noticed in Gardner’s reading is that it overlooks two key (and interrelated) factors: (1) the significance of the ontology of *structure* developed in *The Structure of Behavior* (1942/1963) for understanding Merleau-Ponty’s overall project^20^ and (2) how Merleau-Ponty in the *Phenomenology* limits and transforms transcendental philosophy with his understanding of the phenomenological method. In the next section, I elaborate on the first of these, before I move on to discuss Merleau-Ponty’s methodology in *Rethinking Transcendentalism: the Limits of Transcendental Reflection*.

## Rethinking Nature: Structures of Behavior^21^

What is the “ontology of structure?” Most generally, it is a view of consciousness as an *embodied and expressive* mode of existence that is ontologically prior to the subject–object dichotomy. As Merleau-Ponty puts it in *Structure*’s preface,

taken in itself, [the notion of behavior] is neutral with respect to the classical distinctions between the ‘mental’ and the ‘physiological’ and thus can give us the opportunity of defining them anew. […] By going through behaviorism […] one gains at least in being able to introduce consciousness, not as psychological reality or as cause, but as *structure* (1963, pp. 4–5).

The most important implication of this view for our purposes is that neither the third-person approach of science nor the first-person approach of transcendental phenomenology alone can claim privileged access to the being of consciousness. How does this follow? While I will not be able to give a full account of Merleau-Ponty’s ontology of structure here, I will point to a couple of key elements that motivate this conclusion. Let us start by considering a claim Merleau-Ponty makes with regard to what he calls “vital forms,” the kind of structure of behavior paradigmatic of non-human animals:^23^

the reactions of an organism are understandable and predictable only if we conceive of them, not as muscular contractions which unfold in the body, but as acts which are addressed to a certain milieu, present or virtual: the act of taking a bait, of walking toward a goal, of running away from danger (1963, p. 151).

Trivial as it may seem, this observation is of crucial philosophical importance, in the sense that it is a clear illustration of the above-mentioned diffusion of the dichotomy between the subjective and objective. What it says is that, even when approached from a third-person perspective, the behavior of living organisms is expressive of what we might call a “subjective dimension,” in the form of displaying a relationship to the world as significant *for* the organism in question. Put differently, Merleau-Ponty is here describing a phenomenon where the “internal” (significances *for* the organism) is expressed in the “external” (observable behavior). The “subjective” or “internal” as understood here is thus not some kind of “extra” feature added upon purely objective movements; it is their structure, or *form*, and as such, as I will say more about soon, it is integrated with its “parts” in a relationship of codeterminacy. At its core, the ontology of structure is the view that this embodied-expressive *integration* of subjectivity and objectivity (or the first and third person) is consciousness’ primordial mode of existence and hence the ground from which the notions of the mental and the physiological are abstracted.^24^

Now, admittedly, the claim in the above quote is not so much ontological as it is epistemological, i.e., it is telling us how organisms’ behavior must be *conceived* in order to be understandable, rather than establishing that organisms ultimately *are* one way rather than another. Here, then, we are confronted with a challenge to my claim above that the subjectivity exhibited by organisms’ behavior is not an additional, separable feature—for, can it not be the case that the “sense” we see in living behavior is merely a result of our projections as observers?^25^ This challenge is a decisive moment in the dialectic toward the Integrationist View, motivating, as it does, a return to the position of the philosopher or scientist *qua* the subject seeking to understand consciousness’ place in nature: Can it not be the case that the sense displayed in the behavior of living organisms is merely the result of *our mode of understanding or perceiving*, and not something that is “really there,” in nature? This is the cue for the transcendental philosopher to step onto the stage: the focus have now shifted from the nature of mind and life to how the phenomena of structures of behavior—in this context, vital forms—are constituted as phenomena *for* consciousness. The perspective on consciousness we have entertained so far in this section has been *transcendentally naive*—it has been that of an “external spectator” leaving its own status *as* spectator unquestioned. As many, including Merleau-Ponty himself, have noted, this is the perspective from which most of *Structure* is written^26^; only in the last chapter of that book do we see a shift begin to take place toward a “transcendental” perspective. Let us, however, leave the execution of this shift on hold for a moment, while we let the naive spectator provide us with some more flesh on the bone of the ontology of structure.

The ontology of structure consists in taking the organism as a whole, in its dynamic interactions with its environment, as an irreducible “unit” of nature. Irreducible, because the existence and function of any smaller “part” of this unit (such as physiological features) depends on it being a part of this greater whole, just as the whole in turn depends for *its* existence on the existence and functioning of its parts. As such, structures of behavior are characterized by what Evan Thompson labels “dynamic co-emergence,” meaning “that a whole not only arises from its parts, but the parts also arise from the whole. Part and whole co-emerge and mutually specify each other” (2007, p. 38). While this sort of part–whole relationship can be found also in some non-living physical structures, the structure of living beings is further characterized by having an equilibrium that depends upon “virtual” conditions—that is, conditions produced by the organism itself, and which hence do not exist independently of it (Merleau-Ponty, 1963, p. 145). In Thompson’s terminology, living organisms are *autonomous* systems (2007, p. 37)— systems that themselves generate and maintain the processes necessary for continuing their existence as such. As a concrete case in point, consider the way in which your existence, as a bodily being, is generated by metabolic processes that in turn are maintained only insofar as you interact with your surroundings in a certain way—seeking food when you’re hungry, safety when you’re scared, and so on. Neither these vital significances of your external world (“food,” “safety”) nor the metabolic processes of your cells are things that can exist independently of you as a holistic structure of behavior—they are brought forth *by* this structure while simultaneously being among the conditions necessary for the maintenance of the same structure.

There are two main reasons for why these points regarding the autonomous and dynamic coemergent nature of structures of behavior are important for our purposes. First, they provide us with a helpful framework for making sense of what I earlier called the “subjective dimension” of living structures. Second, they enable us to see how consciousness, *qua* dynamically coemergent structure, is *vulnerable and contingent—*a point that is key to understanding how the Integrationist View sees the relationship between phenomenology and empirical science.

Starting with the first of these, consider how the notion of autonomous systems accounts for the existence of the three interrelated phenomena of (1) selfhood or individuality, (2) a world or environment with a certain relevance or sense *for* the system, and (3) normativity concerning the system’s state and interactions.^27^ Through generating and maintaining itself, the system produces itself *as* self or individual by distinguishing itself from its surroundings. By way of the same process, the surroundings gain a sense or relevance for the system in light of its project of self-generation and self-maintenance. Given that a certain functioning both of the system’s internal organization and of its interactions with its surroundings are of literally existential significance, the emergence of autonomous systems is simultaneously the emergence of a form of normativity pertaining to the system in question; certain states and interactions are more *preferable* than others for the organism in light of its project to keep on existing. In short, a living organism’s structure of behavior is expressive of a network of relations (of dependence, interests, understanding, etc.) between the organism as individual and its environment, brought forth by and meaningful in light of the self-concern of the organism as a whole. This is what accounts for the “subjective dimension,” or form, of living organisms’ behavior.

As hinted above, a crucial consequence of this view is that it amounts to what we might call a “deprivatization” of consciousness. That is, as understood here, consciousness resides in embodied-expressive behavior and is as such not exhausted by its first-person access to itself but is publicly available. This view is expressed by Thompson when he says that

The intentional arc and being-in-the-world overall are neither purely first-personal (subjective) nor purely third-personal (objective), neither mental nor physical. They are existential structures prior to and more fundamental than these abstractions (2007, p. 248).

“The intentional arc” denotes the network of relations between the living organism (or subject) and its world or, in other words, the ways in which the former is situated in and directed toward the latter. To conceive of the intentional arc as an existential structure is to give up on the idea of consciousness as an essentially “inner” mode of being. “The mental,” as Merleau-Ponty puts it in *Structure*, “is reducible to the structure of behavior” and “[s]ince this structure is visible from the outside and for the spectator at the same time as from within and for the actor, another person is in principle accessible to me as I am to myself […]” (1963, pp. 221–222). This view is carried on even after the shift from *Structure*’s “spectator perspective” to *Phenomenology*’s “internal” study of consciousness, where Merleau-Ponty already in the preface echoes the citation from *Structure* in stating that

I must be my exterior, and the other’s body must be the other person himself. […] my existence must never reduce itself to the consciousness that I have of existing; it must in fact encompass the consciousness that *one* might have of it, and so also encompass my embodiment in a nature and at least the possibility of an historical situation (2012, lxxvi).

In other words, my existence as consciousness is not limited to my first-person perspective but extends beyond it. Importantly, this should not be understood as an elimination of first-personal experience or a rejection of the idea that each individual enjoys a special sort of “access” to his or her lived experience that is unavailable *as such* to others. The point is that my reality as subject exceeds what I can grasp through my own perspective upon myself, which means that I am not the sole authority when it comes to understanding my own existence. This deprivatization of consciousness suggests a rethinking of transcendental philosophy, which will be further explored in the next section: If the transcendental is the structural organization in virtue of which stuff appears to consciousness in the first place, and consciousness is an existential structure not exhausted by the first person but visible from “the outside,” then it seems plausible that transcendental reflection must incorporate this “external” view upon consciousness in order to be adequate.

The second reason mentioned above, concerning the contingency and vulnerability of structures, points in the same direction. If consciousness is an existential and bodily structure characterized by a relation of dynamic coemergence between parts and whole, there seems to be little room for the traditional transcendental trait of *a priori necessity* in its organization. On the contrary, being dependent on the proper functioning of its parts for the maintenance of its mode of existence, the structure of consciousness seems to be susceptible to significant reorganizations in reaction to empirical events. Consider, for instance, the case of pathology. It is tempting to understand pathological subjects on the model of a “normal” human way of being, thematizing the illness as a lack or distortion of individual features or capacities relative to this standard. As we will see in the last section of this paper, however, pathology is better understood if we acknowledge that illness, as Merleau-Ponty puts it, “is a complete form of existence” (2012, p. 110). As such, pathology is not so much a case of absent or disturbed particularities relative to an otherwise intact “normal” structure, as it is a different way of existing altogether, a novel, albeit disintegrated, way of being situated in and directed toward the world.^28^

The takeaway for now is this: Considered as existential structure, consciousness is both “deprivatized” and fundamentally contingent. Both of these seem to suggest that there is an important role for empirical science to play together with phenomenology in illuminating the structures of consciousness. After all, empirical perspectives are required in order to adequately grasp the contingencies of our embodied existence, describing, for instance, how humans’ way of relating to the world is affected by bodily injury and traumatic experiences, or what role empirical matters play in childhood development. The question, however, is whether the above reflections have any bearing on the transcendentalist challenge to naturalized phenomenology.

While I think that the ontology of structure in the end will prove to offer what MT lacks (i.e., a framework for understanding how the transcendental subject and the empirical subject can be the *same* subject approached from different perspectives), I am not under the illusion of having convinced the transcendental philosopher yet. After all, recall that the conception of consciousness as structure of behavior that has hitherto been developed is based on the transcendentally naive perspective of an “outside spectator” taking for granted the way in which his or her access to phenomena is constituted or achieved in the first place. Even though I have been advocating a conception of consciousness as an embodied-expressive structure integrating subjectivity and objectivity, thus challenging standard “objectivist” views of nature, this model is still that of consciousness as an *object in the world*, as a phenomenon *for me* as perceiver and thinker. What the transcendental philosopher is concerned with, remember, is not consciousness as object but rather as our *access* to objects as such. This is probably the explanation for why Gardner so quickly dismisses the significance of Merleau-Ponty’s first work in the context of determining his stance toward transcendental philosophy.^29^

There are, however, at least two reasons why this dismissal is too quick. First, it seems possible to argue that the above-mentioned elements of the ontology of structure are in fact relevant to the transcendental project. After all, what I have tried to outline here is a rethinking of nature^30^ that acknowledges a dimension of being prior to the subject–object dichotomy, where consciousness is reconceived as essentially manifest in the grammar of behavior, and our bodily being is seen as organized toward an environment of meaning. Thus, although the ontology of structure might have its origin in a third-personal perspective, its ultimate consequence is a diffusion of the distinction between first- and third-personal perspectives—organisms’ perceived, meaningful environment is exhibited in the behavior by which it is enacted, and must be incorporated as such by the (“third-personal”) scientist in order to be adequately understood. Since observable bodily behavior in this way expresses the constitution of an environment of meaning, it seems possible that it also bears clues of transcendental significance, disclosable by scientific perspectives. In the next section, we will see how this transcendental significance of the notion of structure is motivated also by the “internal” perspective of the *Phenomenology*.

Second, and on a more scholarly note, Gardner seemingly ignores how Merleau-Ponty in the last pages of *Structure* sets the stage for the *Phenomenology’s* transcendental project. That is, there is a tension running through *Structure* that is brought to the fore in its last chapter and which seems to be the motivation for at least some core parts of the *Phenomenology*. This tension is related to the challenge mentioned earlier, regarding the relation between the contributions of our mode of understanding and the embodied-expressive *sense* characteristic of the holistic structure of living organisms’ behavior. The problem, as Toadvine notes, is that the ontology of structure is based upon how the behavior of living beings *appears* as meaningful wholes to a subject, thus giving “the impression that [it] involves a return to idealism, since every structure would have consciousness as its essential correlate” (2009, p. 38). We are thus confronted with the possibility that the sense we disclose in nature belongs only to nature *for us*, that it is a product of our human significations.

Merleau-Ponty’s diagnosis of this problem points directly toward his project in the *Phenomenology*. What leads us toward the idealistic conclusion is that we identify structures with *significations* dependent upon our human conceptualizing capacities, thus privileging, as Toadvine puts it, “the perspective of intellectual consciousness” (ibid.) as our access to the world. The problem can be avoided, however, if we acknowledge that intellectual consciousness is derivative from *perceptual* consciousness, and hence “return to perception as a type of original experience in which the real world is constituted in its specificity” (Merleau-Ponty, 1963, p. 220). This call for a *phenomenology of perception* is made in explicit opposition to traditional, Kantian transcendental philosophy, which sets the contribution of the categories of the understanding center stage. Given that this intellectualist theory is not acceptable, Merleau-Ponty famously states in *Structure*’s very last page, “it would be necessary to define transcendental philosophy anew in such a way as to integrate with it the very phenomenon of the real” (1963, p. 224). Here, we have a clear formulation of the path forward for Merleau-Ponty’s thought, i.e., to *redefine* transcendental philosophy in a way that does justice to the reality of structures. While the shift from intellectual to perceptual consciousness, which Gardner too acknowledges, is a crucial part of this redefinition, an equally important factor is the way in which this entails a methodological integration of phenomenology with scientific perspectives. How are we to understand this methodology?

## Rethinking Transcendentalism: the Limits of Transcendental Reflection

The *Phenomenology* immediately picks up the thread from *Structure*, addressing the nature of the methodology that is to be employed in its “return to perception.” Thus Merleau-Ponty starts, in the very first sentence, with the question “What is phenomenology?” (2012, p. lxx). According to Gardner, Merleau-Ponty in his response “*avows a commitment to* phenomenology conceived as ‘a study of essences,’ ‘a transcendental philosophy,’ ‘a rejection of science”’ (2015, p. 304; my emphasis), thus confirming his own transcendentalist reading. Looking at what Merleau-Ponty is actually saying in the relevant passage, however, this is a far too strong claim, highlighting only one side of what is really presented as *tensions* found within phenomenology. That is, the claim that phenomenology “is the study of essences” is immediately followed by the qualification that “yet [it] also places essences back within existence and thinks that the only way to understand man and the world is by beginning from their ‘facticity”’ (2012, p. lxx). Furthermore, while phenomenology is “a transcendental philosophy […] *it is also* a philosophy for which the world is always ‘already there’ prior to reflection” (ibid.; my emphasis). Lastly, after stating that phenomenology attempts to describe experience “such as it is, without any consideration of its psychological genesis or of the causal explanations that the scientist […] might offer of that experience,” Merleau-Ponty points out that in the last works of Husserl one also finds the notion of a “genetic phenomenology” (ibid.). Ending the paragraph by referring to these as “contradictions,”^31^ it is clear that he is here not *avowing a commitment* to a specific conception of phenomenology as much as he is, as Reynolds puts it, acknowledging “a constitutive methodological disunity at the heart of phenomenology” (2017, p. 87). Rather than providing an answer to the initial question, then, Merleau-Ponty is here offering a further elaboration of the *difficulty* of providing such an answer.

Given the significance of this “methodological disunity” for understanding the nature of Merleau-Ponty’s project, it is surprising that the question of the phenomenological method is not addressed at all in Gardner’s paper.^32^ If Merleau-Ponty is a transcendental philosopher, then by what means does he access the transcendental domain? Here, his claim, which we briefly touched upon in *The Transcendentalist Challenge and Varieties of Transcendentalism*, that “the most important lesson of the reduction is the impossibility of a complete reduction” (2012, p. lxxvii) becomes relevant. What does it mean?

The way to make sense of this at first glance enigmatic statement is to see it as signaling an immanent critique of transcendental philosophy, in the sense that it represents a case of turning transcendental reflection against itself. That is, starting from the position of the transcendental philosopher aiming to fully grasp the constitutive structures of the world’s presence for consciousness, we discover a resistance to our endeavor that ultimately turns out to be an unsurpassable limitation for our project, namely, the fact that we are situated and inextricably involved in a world in ways that can never be exhaustively conceptualized. This, in short, is the fact of embodiment, the concrete, perspectival nature of our existence that makes presence always come at the expense of a certain absence, most simply exemplified by how the visual presence of objects is characterized by the absence of the sides not facing us. The general point here is that, due to our situated, bodily nature, any act of bringing something into view, of achieving presence, or of thematization, is enabled by a background that is “out of view,” absent, or unthematized. From our position as transcendental philosophers, this is obviously a problem: It entails that our reflection, which aims to illuminate the enabling conditions of experience, itself depends upon conditions that it *cannot* fully thematize.

In other words, what we learn from the reduction is, negatively, that the presence of the world *resists* our attempt to reduce it to something that can be exhaustively thematized in acts of reflection and, positively, that consciousness is characterized by a primordial and inescapable bond to the world, which is presupposed by all of our more intellectual mental activities. Thus, the impossibility of a complete reduction has implications for our understanding of both the methodological status of phenomenology as well as the ontological status of consciousness.

First, note that the assumption that a complete reduction is *possible* itself rests upon an unquestioned, naive presupposition, namely, that of a subject enjoying full reflective access to the structures constitutive of its openness to the world. Furthermore, the thought that the execution of the *epoché* can provide this sort of access seems committed to the belief that the meaningful presence of the world is reducible to a meaning for consciousness *qua* reflecting subject. Thus conceived, the phenomenological reduction would, as Merleau-Ponty remarks, “be idealist, in the sense of a transcendental idealism that […] strips the world of its opacity and its transcendence” (2012, p. lxxv). This description, I think, fits the sort of idealism—which mistakes perceived form for intellectual significance—that was at the root of *Structure*’s tension concerning the ontology of structure. What we are seeing here, then, is an internal critique of that view: Taking the possibility of its own project for granted, transcendental reflection’s search for the presuppositions of experience is blind to *its own* presuppositions. In other words, it neglects that we are not constantly reflecting subjects, but that reflection has a *beginning*, and as such is “a genuine creation, a change in the structure of consciousness […]” (ibid., p. lxxiii). The task thus becomes one of uncovering the origin of reflection and the unreflective ground from which it arises.

This is the task of what Merleau-Ponty calls “radical reflection” (ibid., p. lxxviii), which, in Toadvine’s words, is a reflection that “aims to take into account its own immemorial past, its pre-reflective life in nature, as the fundamental condition for its operation as reflection” (2009, p. 53). This, then, is the method of Merleau-Ponty’s novel form of transcendental philosophy, distinguished by its aim to uncover the genesis of reflection rather than taking it for granted. How can this be done? I think Morris is on to something when he observes that “who we are as reflectors […] is a much more contingent and empirical question than the naïve [intellectualist] view would allow” (2018, p. 85). In order to see how that is so, consider the ontological implication of the discovery of the impossibility of a complete reduction. Leading us to recognize our inextricable entanglement with the world as embodied beings, the assessment of a complete reduction as impossible is a first step toward establishing “from the inside” what *Structure* did “from the outside;” namely, that the being of consciousness is primordially that of an embodied structure of engagement with and situatedness within a world or environment, not fully graspable from this structure’s “subjective” point of view. This is another essential turning point in the dialectic toward the Integrationist View: The objection that the notion of consciousness as structure of behavior is transcendentally naive is here countered with the observation that the reflecting activities of transcendental philosophy *themselves presuppose the philosopher’s existence as structure*. In short, just like an adequate third-person understanding of living organisms presupposes recognizing the “subjectivity” displayed in their behavior, an adequate first-person understanding of subjectivity presupposes recognizing it as integrated in a living body’s “deprivatized” and contingent mode of existence as structure.^33^

Let us revisit the phenomenon of contingency now that we have established the connection between transcendental philosophy and structures of behavior. Transcendental philosophy, remember, usually aims to identify the necessary, constitutive structures of experience. Given what we saw in the previous section, regarding the fundamental contingency of consciousness understood as structure, can we still talk about any sort of necessity pertaining to its organization? We might, in this sense: For any mode of existence, there will be processes, structures and features that are necessary for it to maintain its specific way of being situated in and directed toward the world *in the way that it currently is*. That is, as a dynamically coemergent structure, every aspect of our embodied being is in some sense necessary for our holistic form of existence to remain as it is. Being dependent upon empirical contingencies and as such vulnerable to change, however, such “necessary” features of consciousness understood as structure are necessary only in a limited, relative sense. This, I take it, is Merleau-Ponty’s point when he states that

It is impossible to distinguish in the total being of man a bodily organization that one could treat as a contingent fact and other predicates that necessarily belong to him. Everything is necessary in man, and, for example, it is not through a simple coincidence that the reasonable being is also the one who stands upright or who has opposing thumbs—the same manner of existing is expressed in both of these cases. And everything is also contingent in man in the sense that this human way of existing is not guaranteed to each human child through some essence acquired at birth […] (2012, p. 174).

This unorthodox conception of necessity—which refers to the constitutive, yet contingent form of embodied human existence—seems to include any detail that contributes to our total way of being in the world as such. Ultimately, if put “back into my living body” (i.e., if seen as parts of my holistic embodied-expressive existence) even “my ears, my nails, and my lungs […] will no longer appear as contingent details” because “[t]hey are not indifferent to the idea of me that others form, they contribute to my physiognomy or to my style” (2012, p. 455). “Physiognomy” and “style” here refer to my existence as embodied-expressive structure. Notice that this appeal to how I am present to *others* is in line with the above-mentioned deprivatization of consciousness—my existence as subject is not exhausted by my first-personal access to myself but comprises my existence as appearance in the world, available to other perspectives.

It is difficult to see how this notion of necessity we have discovered here is compatible with Gardner’s claim that “the conditions that [Merleau-Ponty’s] phenomenology uncovers are intended to be in the true and genuine sense transcendental, i.e., *a priori* and necessary, and non-identical with empirical, contingent, or mundane states of affairs” (2015, p. 300). On the contrary, what we seem to have now is an *integration* of necessity with contingency, in the sense that the “necessity” pertaining to consciousness as structure is merely its holistic organization of contingent details on which it in turn depends.^34^

If the transcendental has now become the holistic organization of a deprivatized and contingent consciousness *qua* existential structure, then the idea of transcendental philosophy as completely indifferent to scientific matters seems hard to defend. That is, if we want to understand how reflection can arise from our existence as structure, we have to involve perspectives that can illuminate structures of our being not accessible from our point of view as self-reflecting philosophers.

Let us return to the position of the first-person phenomenologist in order to get a better grip on the task at hand. While the phenomenological reduction, as we have seen, cannot consist in a full bracketing or suspension of our attitude toward the world around us, it is nonetheless a productive undertaking in the sense that it loosens “the intentional threads that connects us to the world in order to make them appear” (2012, p. lxxvii). In other words, the reduction enables us to appreciate the complexity of our dependence on and directedness toward the world—the complexity, that is, of the intentional arc. What is thus revealed is a field of preobjective, indeterminate, or *ambiguous* phenomena—the phenomenal, or *transcendental*, field. This field is transcendental in the sense that it is the always presupposed ground for our thoughts and reflections. As we saw in *The Transcendentalist Challenge and Varieties of Transcendentalism*, ascribing this role to a *preobjective field* represents a significant shift from traditional transcendental philosophy. Furthermore, the preobjective nature of the transcendental field means that, while being an enabling factor *for* reflection, it is never fully graspable by the objectifying acts *of* reflection. The word “field,” says Merleau-Ponty, “signifies that reflection never has the entire world […] spread out and objectified before its gaze, that it only ever has partial view and a limited power” (2012, p. 62). The reflecting subject inevitably finds herself always already situated *within* the phenomenal field, presupposing this unreflective bond in all acts of reflection, in the sense that thought only ever gets started against the background of something *unthought*. The phenomenal field, then, is fundamentally *ambiguous* or *indeterminate*, since it resists reflection’s demands for determinacy and clarity by always escaping its full grasp, lending itself to an indefinite number of alternative—perhaps conflicting—acts of determination.

The task of radical reflection is to illuminate this field and the intentional arc that sustains it, with the aim to understand how reflection emerges in the first place. What is clear from what we have seen above is that a philosopher’s reflections alone are not up for this job. Given the complexity of the intentional arc, which “ensures that we are situated within […] our past, our future, our human milieu, our physical situation, our ideological situation, and our moral situation” (Merleau-Ponty, 2012, p. 137), this task is multifaceted and demands a variety of approaches. This, I think, is how we should understand the significance of Merleau-Ponty’s extensive engagements with empirical science throughout his *oeuvre—*that is, as integrating these non-philosophical perspectives into his project of radical reflection. Note that this is a direct contradiction of Gardner, who reads Merleau-Ponty as ultimately reaching a purely transcendental domain, strictly independent of scientific considerations. What we have seen above, on the contrary, is that the idea of transcendental reflection as autonomous and independent from anything other than itself is doomed to neglect its own emergence as reflection and hence fail to adequately perform its task, left rather to spin in a frictionless void^35^ of its own creation. As *radical*, transcendental reflection acknowledges its own limitations and seeks to incorporate a plurality of perspectives in its project of uncovering the ground for its own genesis in contingent and deprivatized existential structures of behavior. Thus, rather than being opposed to all efforts of “naturalization,” a genuine transcendental phenomenology rather *requires* an integration of phenomenological and scientific perspectives.^36^

“Integration” is here not meant to entail complete alignment of the transcendental with the scientific in all respects—after all, their aims are often distinct, and communication between them, while desirable, is never guaranteed. What it means is that they are in a relationship of “interpenetration,” as Merleau-Ponty would say: Each have the potential to gain something from the other and should be pursuing this possibility given the fact of their common origin and their participation in the same pluralistic field of nature. Admittedly, this claim remains empty as long as we have not seen such an integration actualized on a concrete case. This is perhaps the most important implication of the Integrationist View: It is actualized *in* integration, in the sense that it is first in engagement with the concrete and particular that its content is adequately articulated. Thus, let us finally turn to see how Merleau-Ponty’s engagement with the Schneider case is an example of Integrationist naturalized phenomenology in practice.

## Integration in Action: the Schneider Case

The scientific research that figures most extensively in the *Phenomenology* is Adhémer Gelb’s and Kurt Goldstein’s studies of neurological pathology in World War I veterans, in particular their observations of the patient called “Schneider,” who had been struck by shrapnel from a mine to the back of his head, causing severe injury to his brain.^37^ Although Merleau-Ponty engages with various aspects of the Schneider case throughout several chapters of the *Phenomenology*, I will here limit my discussion to Schneider’s pathology as it is presented in Part 1, Chapter III, “The Spatiality of One’s Body and Motricity.” There, the main concern is the relation between “abstract” and “concrete” movements. A distinction between these forms of movements is suggested by the fact that Schneider is unable to point to areas of his body when asked to do so, although he is perfectly able to grasp or touch the same areas if those movements are called for by the immediate, concrete situation, for instance when bitten by a mosquito (2012, p. 106). This distinction is applicable also to another curious abnormality displayed by Schneider: Although he performs the tasks of his work without difficulty when in the actual situation of his working place, having the required instruments at hand, he is unable to *imitate* the same movements without elaborate preparations, having to, so to speak, actively “place” his whole body virtually within the concrete situation of his working place (ibid, p. 112). Similarly, he is unable to move his hand into a military salute without assuming a whole military-like posture, producing the concrete situation where such a movement is called for (ibid.).

What we have here is a collection of empirical descriptions of patterns of pathological behavior that emerged after Schneider’s accident. The question now is, what is the significance of these facts for phenomenological philosophy? An appealing yet ultimately too simple way to understand Merleau-Ponty’s dealings with the descriptions of Schneider’s pathology is that he brings a preestablished philosophical framework to bear on a concrete case, corroborating his ontology of structure by showing its supremacy over alternative ways of accounting for the facts. This way of looking at it can be motivated by reconstructing the trajectory of Merleau-Ponty’s thought in the relevant chapter as follows: Revealing the shortcomings of intellectualist and empiricist explanatory strategies, which try to reduce Schneider’s pathology to a malfunctioning pertaining to either the causal processes of the physiological body or to the representational capacities of the mind, Merleau-Ponty shows how Schneider’s disorder can best be understood through the model of existential structures. The problem of whether to account for the disorder as *either* physiological or psychological is thus overcome through the idea of the living body as a structure of behavior: “The motor disorders in cerebellar injury cases and those of psychic blindness can only be coordinated if the background of movement and vision is defined not by a stock of sensible qualities, but by a certain manner of articulating or of structuring the surroundings” (2012, p. 117).

In saying that this way of rendering Merleau-Ponty’s use of the Schneider case is misleading, I do not mean that it is completely false. After all, it is clear that Merleau-Ponty believes his non-reductive, phenomenological approach contributes to a better understanding of Schneider’s pathology. As he says,

Behavior can only be grasped by […] the type of thought that takes its object in its nascent state, such as it appears to him who lives it, with the atmosphere of sense by which it is enveloped, and that seeks to slip itself into this atmosphere in order to discover, behind dispersed facts and symptoms, the total being of the subject (2012, p. 122).

What is misleading is the idea that the relation of enlightenment between philosophy and science here only goes one way, from the former to the latter. In order to see how it rather is a case of *mutual* enlightenment, remember first that the task of radical reflection as staked out in the previous section is to illuminate the structures of its own prereflective ground. Now, how can the study of Schneider’s illness contribute to this project?

Consider the significance of this empirical case as a concrete example of a radical modification of the intentional arc. It is, in other words, an existence proof of the contingency of human consciousness’ situatedness in and directedness toward the world and as such reveals the reality of the integration of the transcendental and empirical.^38^ Schneider’s brain injury initiated a process of a global restructuration of his mode of existence—his motor and cognitive capacities became organized in a new way, took on a new sense, in order to cope with the challenges arising from the damage. Since his pathology concerns his subjectivity as much as his motricity, he no longer has access to the same *phenomenal field* as normal healthy subjects. In other words, the “necessity” of Schneider’s normal human way of disclosing the world was necessary only up until the point a shrapnel hit his head.

Now, as mentioned in *Rethinking Nature: Structures of Behavior* above, this has some important consequences for what we can learn from comparing Schneider with “normal” subjects. As Merleau-Ponty emphasizes, “It cannot be a question of simply transferring to the normal person what is missing in the patient and what he is trying to recover. Illness, like childhood […], is a complete form of existence […]” (2012, p. 110). Just like we cannot understand children’s way of existing as an adult form with certain lacks, the relation between the normal and the pathological subject cannot be understood in terms of subtraction or addition of particular functions pertaining to an otherwise identical structure. Thus, we should not take Schneider’s pathological behavior as exhibiting either the lack or the presence of particular functions that we can then infer are also present in normal subjects. If Schneider’s disorder is not a case of a malfunctioning of any one particular function, but rather the manifestation of a pathological *mode of being*, then the difference between him and normal subjects must be a difference in existential structure—in their global way of engaging with the world.

On this basis, we can use the contrast between Schneider and normal subjects in order to disclose a “transcendental” structure of organization characteristic of the latter’s mode of existence. That is, in considering Schneider’s inability to easily and immediately move his body accordingly in response to instructions even though he understands them intellectually, we are able to catch a glimpse of a structural moment of our prereflective embodied existence that would hardly have been available through the transcendental philosopher’s reflections. Since Schneider, Merleau-Ponty observes, “is missing neither motricity nor thought,” but nonetheless displays this inability to perceive “motor significations,” “we must acknowledge, between movement as a third person process and thought as a representation of movement, an anticipation or a grasp of the result assured by the body itself as a motor power […] or a ‘motor intentionality”’ (2012, p. 113). With this notion of motor intentionality, which is neither a purely mechanical physiological process nor an explicit first-personal thought, we have thus discovered a way to conceptualize our being as embodied structures in a way that was not available to us prior to the empirical case of Schneider’s disorder.

While this might seem as a case of inferring the presence of a feature in normal cases from a lack in the pathological case, the point is on the contrary that motor intentionality has to do with the *total* organization of the normal human structure of behavior.^39^ This is Merleau-Ponty’s point when he argues that “‘visual representations,’ ‘abstract movement,’ and ‘virtual touching’ are only different names for a single central phenomenon” (2012, p. 120), or again, that “visual representations, tactile givens, and motricity are three phenomena cut out of the unity of behavior” (2012, p. 121). One way to characterize the structure of behavior of normal subjects as opposed to that of Schneider is to say, as Merleau-Ponty does, that “the normal person *reckons with* the possible, which thus acquires a sort of actuality without leaving behind its place as possibility” (2012, p. 112). In short, we inhabit a world that, in an important sense, is more *open* than that of Schneider, who, we can say, is “trapped” in an environment that does not offer him the same behavioral possibilities as we have. Thus, motor intentionality—the capacity for immediate bodily grasp of significances—is in the normal case a power that characterizes our total mode of being in the world. In other words, it sustains the *intentional arc*—which, to repeat, is what ensures our situatedness within a complex network of natural and symbolic relations, and further “creates the unity of the senses, the unity of the senses with intelligence, and the unity of sensitivity and motricity”—and it is *this*, Merleau-Ponty claims, which ultimately “‘goes limp’ in [Schneider’s] disorder” (2012, p. 137).

To sum up, if Merleau-Ponty’s analysis is correct, radical reflection has here made progress in uncovering some of the conditions that enable it: Motor intentionality has been established as a transcendental power integrated with contingent embodied life and discovered through engagement with a scientific account of an empirical case. As such, Merleau-Ponty’s engagement with the Schneider case has yielded a result relevant to both science and transcendental phenomenology and is thus a clear case of the Integrationist View in action.

## Conclusion

My aim in this paper has been to propose a response to the Transcendentalist Challenge to naturalized phenomenology by sketching the contours of what I called the Integrationist View. Such a view, I have argued, is required if we want to not only *allow* for a relationship of mutual enlightenment between phenomenology and science (as MT does) but also *make sense* of it. The key to this view is the conception of consciousness as a structure of behavior, ontologically prior to the distinctions between objectivity and subjectivity and third- and first-person perspectives. As we have seen, this “ontology of structure” is in Merleau-Ponty’s philosophy motivated not only by the transcendentally naive perspective of observers of behavior but also equally through an internal critique of the transcendental perspective itself. In this way, we arrive at a view of the transcendental as not essentially separate from the natural, but rather as organizational norms of contingent, living nature that are best illuminated through a dialectical exchange between phenomenological and scientific approaches.

It might be objected that the end result has abandoned transcendental philosophy altogether. Given a certain conception of “transcendental,” that is probably true. However, given the internal critique of transcendentalism involved in IV together with the historical fluidity of transcendental philosophy, I believe the label can be kept if desired.

In the last section, I made an attempt to show a concrete example of phenomenology-science integration, arguing that Merleau-Ponty’s engagement with the Schneider case has the potential to both inform the scientific understanding of pathology and to be a moment in radical reflection’s uncovering of its own conditions. Thus, the integrationist view finally went from abstract articulation toward a concrete *sense*, for, as Merleau-Ponty says in the last page of the *Phenomenology*, “philosophy actualizes itself by destroying itself as an isolated philosophy” (2012, p. 483).

## Author Contributions

The author confirms being the sole contributor of this work and has approved it for publication.

## Conflict of Interest

The authors declare that the research was conducted in the absence of any commercial or financial relationships that could be construed as a potential conflict of interest.

## Footnotes

1 I’m I am here using Gardner as representative for a concern raised by multiple phenomenologists, e.g., (De Preester, 2002; Moran, 2013).

2 Gardner’s examples include reading Merleau-Ponty as providing “a convincing critique of the representationalism which holds sway in cognitive science” and an “account of skill acquisition [that] stands in deep accord with developments in brain science neural network theory” (2015, p. 297).

3 Habermas (1991) provides a clear overview of the development and internal and external critiques of the tradition of transcendental philosophy.

4 This is intended to give a rough grasp—and not in any way to be an exact rendering—of what happens in Kant’s first *Critique*.

5 This is not to say that transcendental philosophy has no interest in science. On the contrary, its main motivation has traditionally been to establish a philosophical ground for scientific knowledge.

6 But see, e.g., Strawson (1966); Henrich (1969), Stern (2000), and Ameriks (2003).

7 For instance, what structures are constitutive of operations in pure arithmetic? (Notice that the question here is neither *whether* it is possible nor whether its results will be universally true). Kant’s answer is that this possibility, among other things, depends on time as a pure, subjective form of intuition (2007, B17, B56).

8 Zahavi (1996) argues convincingly that another way in which Husserl transforms Kant’s transcendental philosophy, is by recognizing the constitutive role of intersubjectivity, thus going beyond the emphasis on the individual consciousness.

9 There are competing interpretations concerning the exact nature of the *epoché* and the reduction in Husserl’s phenomenology. Here, I’m I am relying on Dan Zahavi’s illuminative rendering in *Husserl’s Legacy* (2017, pp. 56–60).

10 The method of eidetic analysis consists, in short, in an imaginative variation of the phenomenon in question in order to become aware of its invariant structure or *essence—*that without which it would no longer be what it is. In this way, it aims to answer questions such as “what do essentially characterize acts of perceiving, imagining, remembering, judging, etc., and how are these different acts related to each other?” (Zahavi, 2017, p. 15).

11 I do, however, not agree that Merleau-Ponty’s transcendentalism warrants the label of an “idealist metaphysics” (2015, pp. 309). I’ll I will not be able to explicitly argue this point here, but I take my case for an Integrationist View of naturalized phenomenology to provide some reasons for rejecting such a description.

12 Although I doubt Gardner’s (2015, p. 297) prime examples of psychological interpreters of Merleau-Ponty—Hubert Dreyfus, Shaun Gallagher, and Sean Kelly—would fit easily into that category.

13 Gallagher’s (2003) idea of “front-loading” phenomenology is a good illustration of how phenomenology can also be used to inform experimental settings.

14 Compare the quote here to Zahavi’s claim in a different text, that “Husserl’s phenomenology is characterized by its attempt to modify the static opposition between the transcendental and the mundane, between the constituting and the constituted” (2010, p. 15). It is outside the scope of this paper to explore whether such a modification is compatible with refraining from “a fundamental rethinking,” but there at least seems to be a tension at the surface here.

15 See, e.g., *The Paradox of Subjectivity* (Carr, 1999).

16 A lot can be—and have been—said about the notions of “nature” and “naturalism” in the context of naturalizing phenomenology. Here, I’m I am limiting myself to a simplified definition, but see, e.g., [Vanzago (2012); Roux (2013), and Reynolds (2018)] for more thorough discussions.

17 As Zahavi (2013, p. 33) has noted, replacing physicalist/reductionist naturalism with an “emergentist” version alone is not sufficient to ease phenomenology’s relation to naturalism, for the latter might still be committed to objectivism.

18 “Mind is life-like and life is mind-like,” as he puts it (ibid., p. 128).

19 “If the promised synthesis of transcendentalism and naturalism could be made plausible independently—no mean feat—then it would furnish the basis for a reconstructive interpretation of Merleau-Ponty, but it is not in Merleau-Ponty’s own line of sight” (Gardner, 2015, p. 318).

20 Admittedly, Gardner does mention the continuity between *Structure* and *Phenomenology* as something that supports the Psychological Interpretation he argues against (2015, p. 296). However, he seems to think that this continuity is only apparent, and limits his attention to *Structure* to a couple of brief remarks describing it as a work of “holist, anti-reductionist thought […] much of which reads like a philosophy of psychology” (ibid.).

21 Kee (2020) too draws attention to the significance of *Structure* for the project of naturalizing phenomenology. As far as I can see, our approaches are largely aligned, although our emphases are somewhat different. One of Kee’s key claims is that a phenomenological reduction is undertaken already in *Structure*’s engagement with psychological considerations, which prompts a shift of attention toward organisms’ perceived world. I do not dispute this, but—as we we’ll see toward the end of this section—I will argue that a more explicit turn toward (transcendental) phenomenology is motivated by a tension that crystallizes toward the end of *Structure*, and that this is what sets the stage for the *Phenomenology*’s project.

23 For the sake of simplicity, I’ll use vital forms to represent the notion of structures of behavior here. It is, however, important to be aware that Merleau-Ponty locates human behavior at a different level of organization from that of non-human animals. In short, human behavior is not merely oriented with regards to vital/biological needs, but is situated within and directed toward an *intersubjective* world with symbolic significances.

24 To avoid any misunderstanding, let me emphasize that the acknowledgment of an “internal” dimension of behavior here means that we, despite the focus on behavior, are going beyond traditional (objectivist) behaviorism. Merleau-Ponty’s point in the above quote is that the objectivist stimulus—response approach of behaviorism—which neglects, as Kee puts it, “the perceived world of the animal itself,” thus failing to recognize “behavior and situation as *internally* related structures with a unique logic” (2020, p. 19)—is unable to adequately understand behavior.

25 Let me note, without being able to argue extensively for it here, that a concession to such a separation between epistemology and ontology is problematic, in the sense that it ultimately leads to an unsustainable skepticism. For someone who challenges the epistemology—ontology separation, see, e.g., Bhaskar (1978, pp. 36–45) or Taylor (1997). Although it is not explicitly addressed in the main text, the claim that there is a sort of meaning *for* living organisms as well as immanent *in* their behavior, is in effect a subscription to teleological view of life. Thus, the epistemological challenge here can be more specifically framed as one of teleonomy (living organisms behave/must be understood *as if* purposive) vs. teleology (living organisms *are* immanently purposive). See Weber and Francisco (2002) for an informative overview of this issue as well as an argument in favor of the latter position.

26 For instance, in one of *Phenomenology*’s footnotes, he distinguishes *Structure* as concerned with consciousness “seen from the outside” from *Phenomenology* as concerned with consciousness “seen from within” (2012, n18 p. 535).

27 Roughly the same points can be found in Thompson (2007, pp. 73–74).

28 In the words of Georges Canguillhem, “disease is not a variation on the dimension of health; it is a new dimension of life” (1978, p. 108). This does not mean that there are no meaningful distinctions to be made between the normal and the pathological, in the sense that the latter cannot be seen as the “worst” of the two. The point is that what is disturbed in the pathological case is ultimately the global behavioral space of the patient, not individual psychological or physiological traits.

29 See footnote 24.

30 Admittedly not nature as a whole, but more specifically in the form of living organisms. However, as Morris (2018) has recently tried to show, this approach to the structure of life might ultimately help us toward a more fundamental rethinking of nature.

31 “Might one hope to remove these contradictions by distinguishing between the phenomenologies of Husserl and Heidegger?” (2012, p. lxx). See Zahavi (2008) for a closer discussion of these remarks from the *Phenomenology*’s preface.

32 As far as I can see, Gardner (2015) mentions the phenomenological method only twice, first in the context of presenting the Psychological Interpretation (p. 296), and later (p. 304) when interpreting a claim Merleau-Ponty makes in *The Visible and the Invisible* (1968). He does, however, never discuss or clarify what he takes this method to be.

33 A key point here is how our self-understanding originates in and depends upon intersubjectivity (intercorporeality), and further how aspects of the self unavailable through self-reflection can be made “visible” through the other’s mimetic responses to my behavior. I will unfortunately not be able to further elaborate this here.

34 Andrew Inkpin draws attention to similar points regarding necessity in the *Phenomenology* (2017, p. 40). He, however, uses this (among other considerations) to conclude that Merleau-Ponty’s project does not warrant the name “transcendental.” While I agree that this is the right conclusion given Inkpin’s criteria for what counts as transcendental philosophy, I think the historical fluidity of this notion makes it possible to rather see Merleau-Ponty as transforming the idea of the transcendental.

35 To borrow McDowell’s (1994) expression.

36 Reynolds makes the same point: “[Merleau-Ponty’s] particular conception of transcendental philosophy […] not only is compatible with a serious and sustained engagement with empirical science but even requires it” (2017, p. 87).

37 Schneider’s illness was initially diagnosed as a case of visual agnosia (Goldstein and Gelb, p. 137), and has later been more narrowly classified as a case of “visual form agnosia” (Farah, 2004, p. 13). I’m I am indebted to Rasmus Thybo Jensen’s paper on Merleau-Ponty’s engagement with the Schneider case for these references (Jensen, 2009).

38 Fernandez makes a similar point in arguing that Schneider motivates the idea of a “contaminated transcendental”: “The *a priori*, ontological structures of the world are contingent precisely because they are contaminated. And the contaminant is the world itself” (2015, p. 296).

39 Jensen (2009) points to an interesting and significant ambiguity on Merleau-Ponty’s part regarding his understanding of the power of motor intentionality. That is, Schneider here seems to be used “in two mutually exclusive ways: motor intentionality is to be revealed both by its perspicuous preservation and by its contrastive impairment in one and the same case” (Jensen, 2009, p. 372). Does Schneider exhibit an intact normal form of motor intentionality, or is rather his illness a case of a distortion of this same function? Given the holistic approach to structures of behavior that I ’ve have advocated above, the idea that *the very same* power of motor intentionality is at work both in normal subjects and in Schneider must be rejected. Rather, if we are to talk about “motor intentionality” in both cases, it must be two different *forms* of motor intentionality—one sustaining a “normal” human organization of existence and one facilitating the maintenance of a more disintegrated, pathological mode of being. While the textual evidence for a contradiction on Merleau-Ponty’s part is surely real, Jensen’s conclusion that “[t]he best way to avoid the contradiction is to accentuate the differences between the concrete actions of the patient and the corresponding actions performed by the normal person […]” (2009, p. 387) supports this reading.
